# Supplementary material for: Alignment and characterization of remote-refocusing systems
Source: Appl Opt. 2023 Sep 25;62(28):7431–40. doi: 10.1364/AO.500281 (PMC10575606; doi:10.1364/AO.500281)
Supplement: Supplementary file 1 [file ao-62-28-7431-s001.pdf]

Supplemental Document

applied optics

Alignment and characterization of remote-refocusing systems: supplement

Wenzhi Hong, Hugh Sparks, and Chris Dunsby*

Photonics Group, Physics Department, Imperial College London, London, UK
**christopher.dunsby@imperial.ac.uk*

This supplement published with Optica Publishing Group on 25 September 2023 by The Authors under the terms of the Creative Commons Attribution 4.0 License in the format provided by the authors and unedited. Further distribution of this work must maintain attribution to the author(s) and the published article's title, journal citation, and DOI.

Supplement DOI: https://doi.org/10.6084/m9.figshare.24100857

Parent Article DOI: https://doi.org/10.1364/AO.500281

Alignment and Characterisation of Remote-Refocusing Systems: Supplementary Material

1. Sample Preparation

A precision #1.5 glass coverslip (630-2186, Marienfield) was cleaned with acetone for 5 minutes, ethanol for 5 minutes and washed three times with water. Then 2 µL 1:100 dilution of poly-L-lysine (P8920, Sigma-Aldrich) in water was incubated on the coverslip at room temperature for 5 minutes. Excess poly-L-lysine was removed by three washes with water. 100 nm fluorescence beads (T7279, TetraSpeck) were diluted 500-fold in water and a drop (50 µL) placed on the poly-L-lysine-coated coverslip for 10 minutes. Unbound beads were removed by washing three times in water. The beads were then covered with polyvinyl acetate (MOWIOL® 4-88 Reagent, 475904 Millipore, Mowiol) and a Superfrost Plus Adhesion microscope slide (J18000AMNZ, Epredia) was placed on top. The coverslip and microscope slide were sealed permanently with silicone sealant (Polycraft ZA22, Mould RTV Addition Cure Mould Making Silicone Rubber, MBFibreglass). The slide was incubated overnight at 37°C to cure the mounting media and silicone.

2. Supplementary Text

2.1 Estimation of Theoretical Diffraction-Limited Lateral Resolution

The estimated ideal point spread function (PSF) is obtained using the convolution of the scalar Airy distribution, a spherical 100 nm diameter bead where the fluorophore is assumed to be uniformly distributed through the sphere, and a square pixel. This can be written as

$$\mathrm{PSF}_{\mathrm{airy\_bead}}(x,y) = \mathrm{Airy}_{\mathrm{ideal}}(x,y) * \mathrm{Bead}_{\mathrm{ideal}}(x,y) * \mathrm{Pixel}(x,y), \quad \text{(S1)}$$

$$\mathrm{Airy}_{\mathrm{ideal}}(x,y) = \{2J_1[(2\pi/\lambda)\mathrm{NA}R]/[(2\pi/\lambda)\mathrm{NA}R]\}^2, \quad \text{(S2)}$$

$$\mathrm{Bead}_{\mathrm{ideal}}(R) = 2(R_{\mathrm{bead}}^2\text{-}R^2)^{1/2} \text{ for } R <= R_{\mathrm{bead}},$$

$$= 0 \text{ for } R > R_{\mathrm{bead}}, \quad \text{(S3)}$$

$$\mathrm{Pixel}(x,y) = 1 \text{ for } |x| <= P/2 \text{ and } |y| <= P/2,$$

$$R = (x^2+y^2)^{1/2}, \quad \text{(S4)}$$

where R_{bead} is the 50 nm bead radius and P is the width of the pixel. This provides an FWHM of 0.23 µm.

The Strehl criterion is used commonly in optics to provide a condition where an optical system can be considered to be practically diffraction limited [1]. A system is considered to be diffraction limited by this criterion if the peak of the actual PSF is greater than or equal to 80% of the PSF that would be obtained if the system was aberration free. To make use of this criterion in the context of the PSF FWHM measured in this work, we considered how much a Gaussian PSF would need to be broadened by (whilst keeping the integrated PSF energy constant) for the peak intensity to drop to 80% of the original value. This results in a broadening factor of $0.8^{-1/2}$. Therefore, the broadened bead FWHM that still meets the Strehl condition is 0.26 µm and we chose to use this to define the region over which the system can be considered to be practically diffraction limited.

2.2 Geometrical Remote-Refocusing Volume

We consider geometrically the volume where rays pass within both the limiting field of view of the system D_{lim} and the limiting angular aperture of the system θ_{lim}, see Fig. S7.

D_{lim} is given by the minimum of the field number of O1, FN_{O1}, and the field number of O2, FN_{O2}, projected into sample space, i.e., $D_{lim} = \min\{FN_{O1}/M_{O1}, FN_{O2}/M_{O2}/M_{total}\}$. For the system used here, D_{lim} = 398 μm, M_{total} = 1.333, and M_{O1} and M_{O2} are the magnifications of O1 and O2.

θ_{lim} is given by the minimum angular acceptance of O1 and O2, i.e., $\theta_{lim} = \min\{\arcsin(NA_{O1}/n_1), \arcsin(NA_{O2}/n_2)\}$. For the system used here θ_{lim} = 64°.

The maximum geometric refocus distance, Z_{max}, occurs when rays emitted at the limiting angle θ_{lim} from a point object on the optical axis away from the objective focal plane just pass through the limiting field of view D_{lim} in the focal plane, see Fig. S7. Using trigonometry, Z_{max} is given by the equation

$$Z_{max} = D_{lim}/(2 \tan \theta_{lim}). \quad \text{(S6)}$$

For the system used here, Z_{max} = 95 μm.

The geometric region over which all rays emitted within the collection cone of the remote refocussing system defined by θ_{lim} is therefore described by a double-ended cone with diameter D_{lim}, tip-to-tip distance $2Z_{max}$ and volume

$$V_{geom} = \pi D_{lim}^2 Z_{max}/6. \quad \text{(S7)}$$

For the system used here, $V_{geom} = 8.0\times10^6$ μm^3. This is in good order-of-magnitude agreement with the values reported below in supplementary Table S4.

3. Supplemental Figures

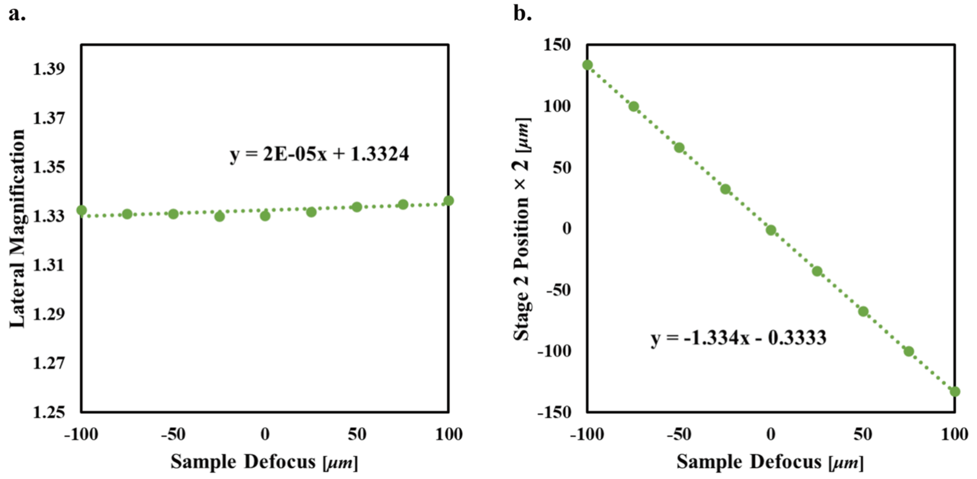

Fig. S1. (a) Linear fit to the measured lateral magnifications within the whole refocusing range. (b) Linear fit to twice the Stage 2 position for the in-focus image as a function of sample defocus. The axial magnification equals to (-) gradient. At each sample defocus, the lateral and axial magnifications were measured from the Stage 2 position closest to the sample being in focus on C2.

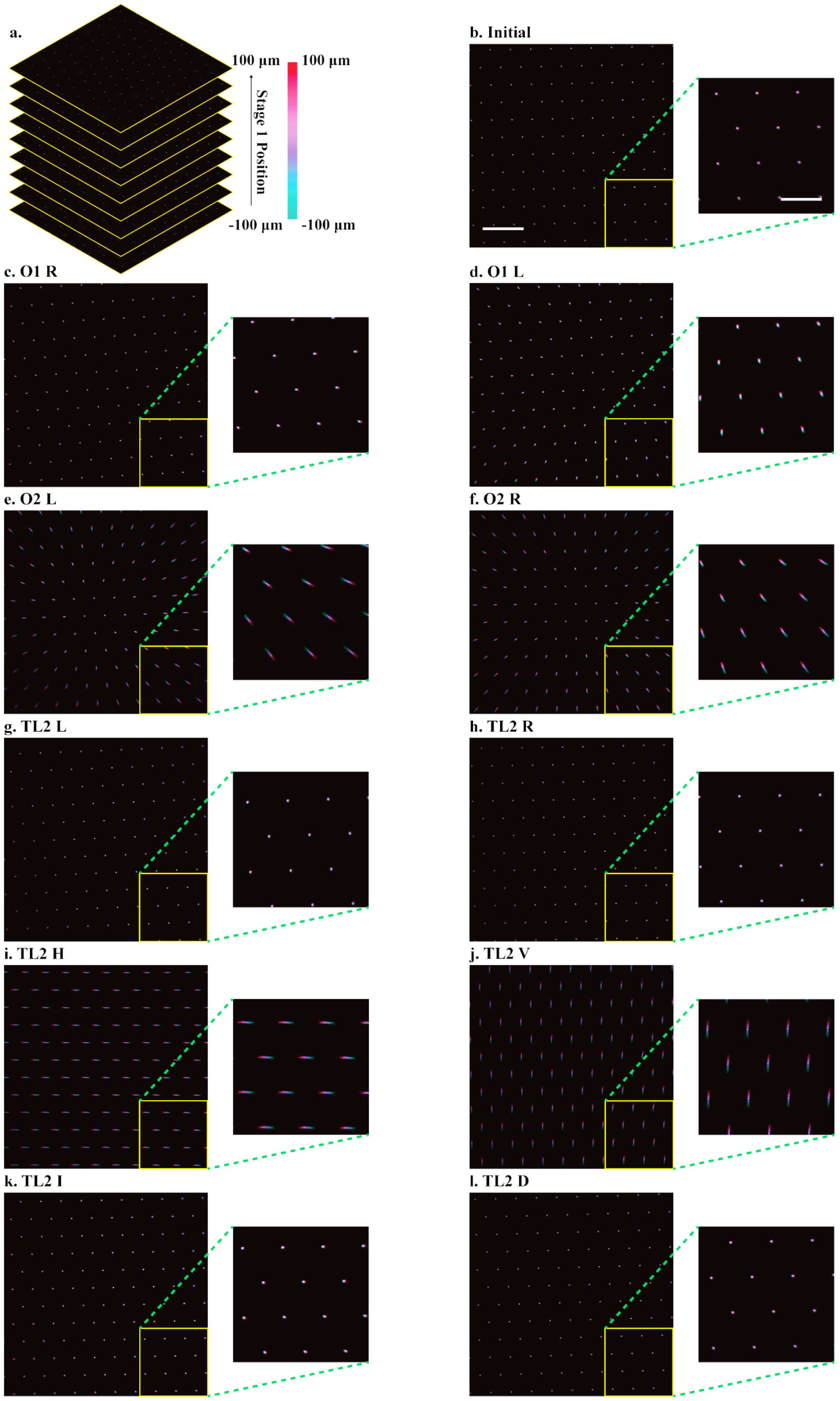

Fig. S2. Color-coded maximum intensity projection (MIP) of 9 in-focus STM images in all tested cases. (a) Cartoon illustrating the 9 in-focus STM images and corresponding axial range and false-colour bar scale. (b-l) Color-coded MIPs across the whole refocusing range for the initial condition (b) and different misalignments (c-l) of the full FOV (2304 pixels × 2304 pixels; scale bar, 40 µm); zoomed-in images on the right of each panel (768 pixels × 768 pixels; scale bar, 20 µm) show the region indicated by the yellow squares. O, objective; TL, tube lens; L, left; R, right; H, horizontal; V, vertical; I, increase; D, decrease. See Fig. 1 for diagram showing directions of motion for each misalignment.

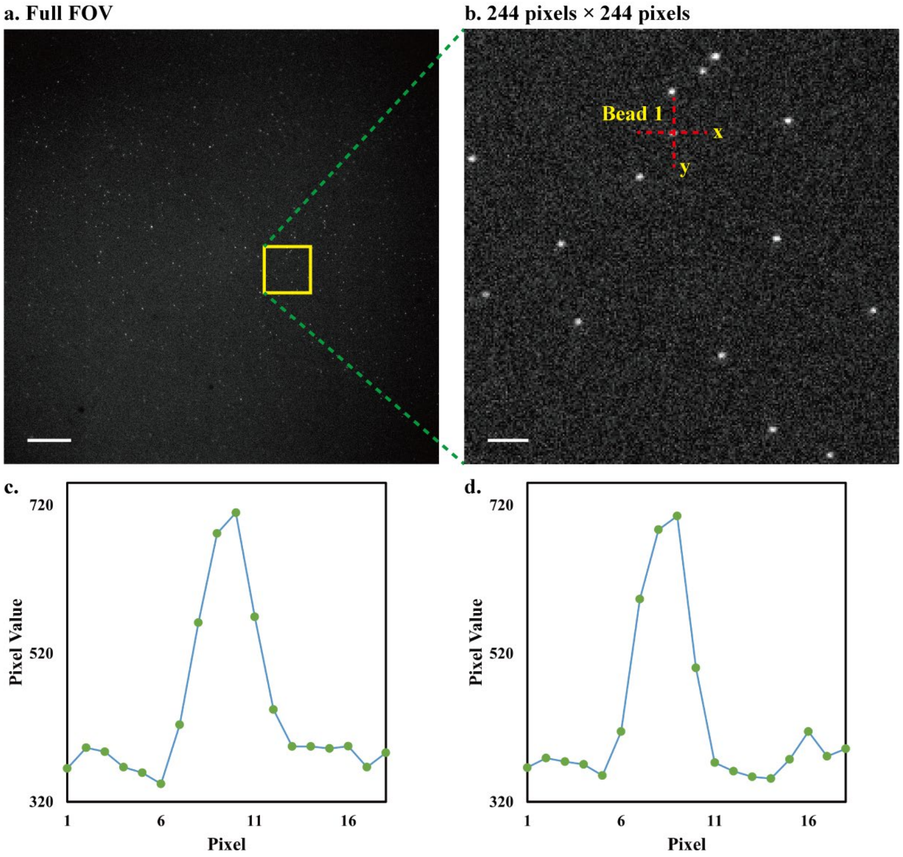

Fig. S3. (a) An example image of 100 nm beads acquired with the initial system at 0 μm defocus position. Scale bar, 20 μm. (b) A zoom-in image of the yellow square in (a). Scale bar, 2 μm. (c) and (d) are respectively horizontal and vertical profiles of Bead 1 indicated with red dashed lines in (b). Gaussian fits to the profiles of beads are shown in blue dashed curves. The FWHM values of curves shown in (c) and (d) are 0.292 μm and 0.273 μm respectively (mean FWHM = 0.283 μm).

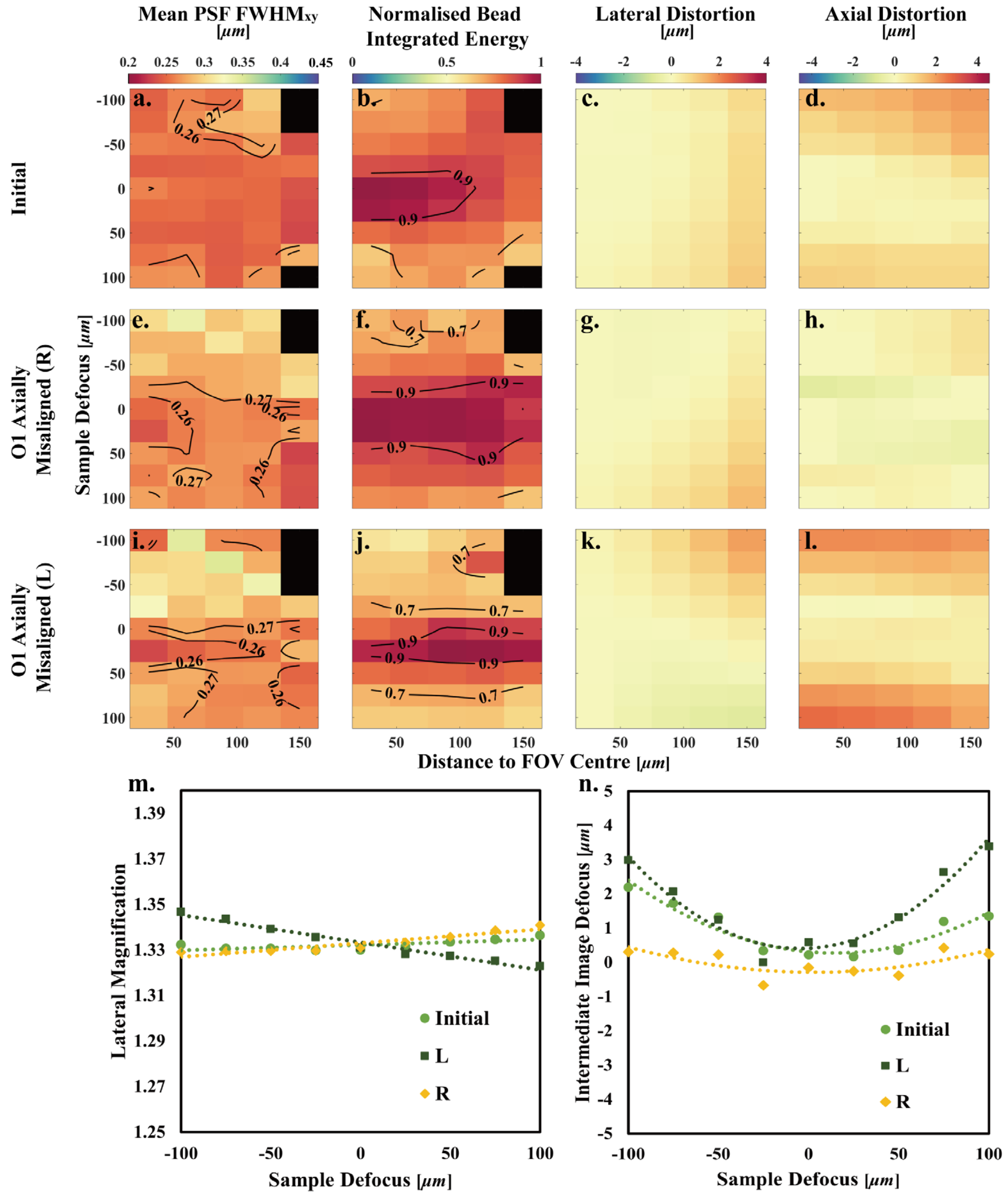

Fig. S4. Characterisation of O1 axial misalignment. (a-d) Initial system. (e-h) O1 axially misaligned by 1 mm towards TL1. (i-l) O1 axially misaligned by 1 mm towards Stage 1. (m) Lateral magnification as a function of sample defocus. The dashed lines are linear fits to the measured data points. (n) Intermediate image defocus as a function of sample defocus. The dashed curves are the second-order polynomial fits to the measured data points.

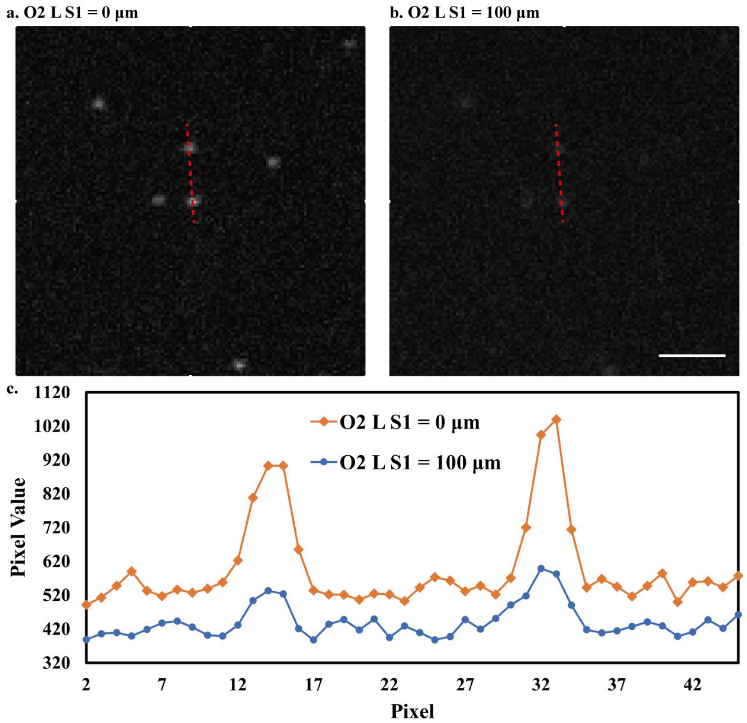

Fig. S5. (a) Image of two 100 nm beads (average 40 µm from the centre of FOV) acquired for misalignment of O2 for a Stage 1 position of 0 µm. (b) Same field of view and conditions except now for a Stage 1 position of 100 µm. In both cases the optical system has been refocused using M2. Scale bar, 2 µm. (c) Line profiles along the red dashed lines indicated in (a) (orange) and (b) (blue). O, objective; L, left; S1, Stage 1.

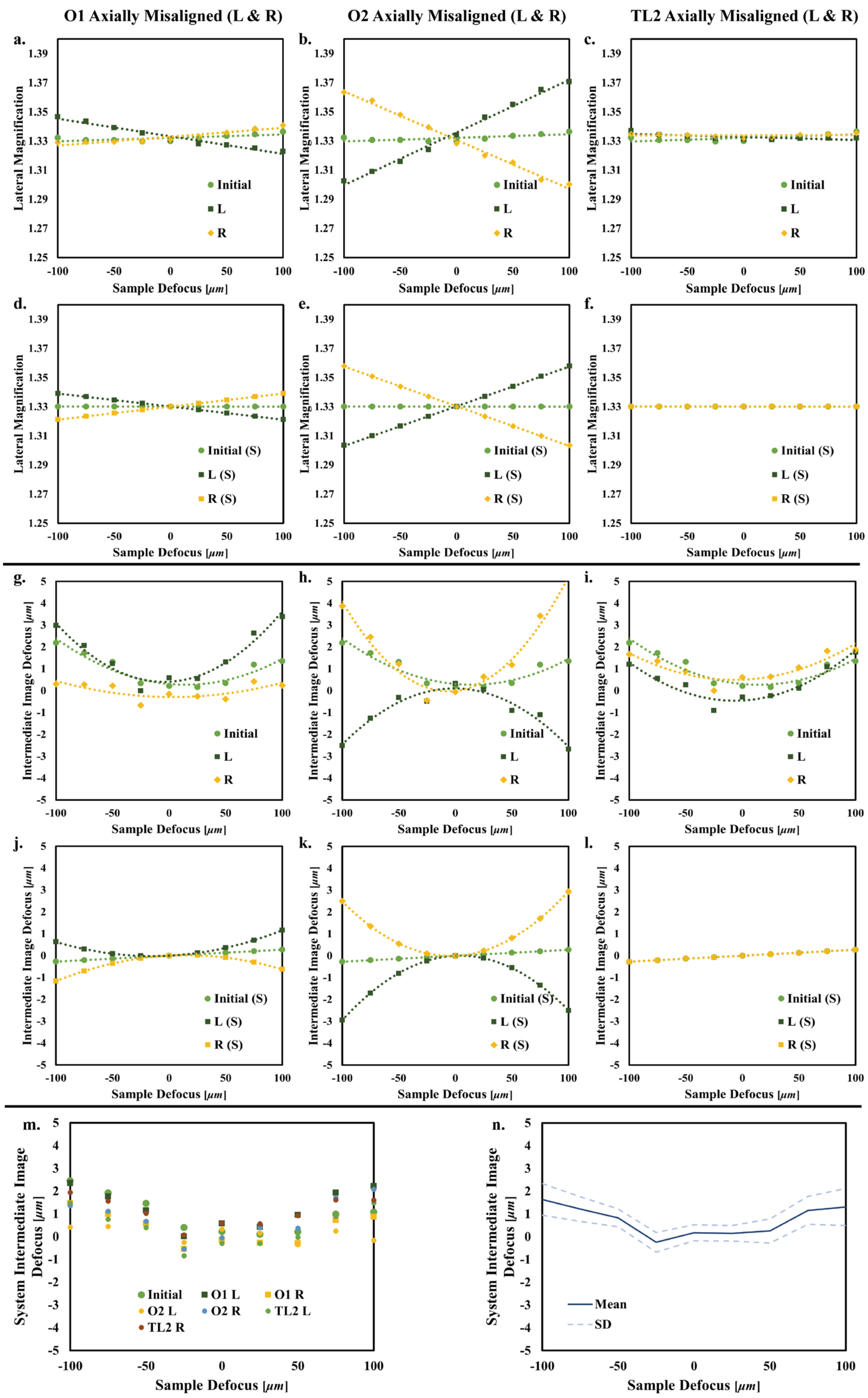

O1 Axially Misaligned (L & R)
O2 Axially Misaligned (L & R)
TL2 Axially Misaligned (L & R)
a.
b.
c.
d.
e.
f.
g.
h.
i.
j.
k.
l.
m.
n.
Lateral Magnification
Intermediate Image Defocus [μm]
System Intermediate Image Defocus [μm]
Sample Defocus [μm]
Initial
L
R
Initial (S)
L (S)
R (S)
Initial
O1 L
O1 R
O2 L
O2 R
TL2 L
TL2 R
Mean
SD

Fig. S6. Comparisons of experimental measurements of lateral magnification and intermediate image defocus with the simulations performed in Zemax using paraxial thin lenses. (a-c, g-i) Experimental measurements of lateral magnification and intermediate image defocus as functions of sample defocus. (d-f, j-l) Simulation results of lateral magnification and intermediate image defocus as functions of sample defocus. (a-c) are the same as figures shown in Fig. S4 (m), Fig. 2 (m) and Fig. 3 (m). (g-i) are the same figures in Fig. S4 (n), Fig. 2 (n) and Fig. 3 (n). (m) Data points obtained by subtracting the simulation results from the corresponding experimental measurements of intermediate image defocus. (n) Average of the values obtained from the different misalignments shown in (m). The two dashed lines indicate plus and minus one standard deviation of the different misalignments in (m). L, left; R, right; S, simulation; SD, standard deviation.

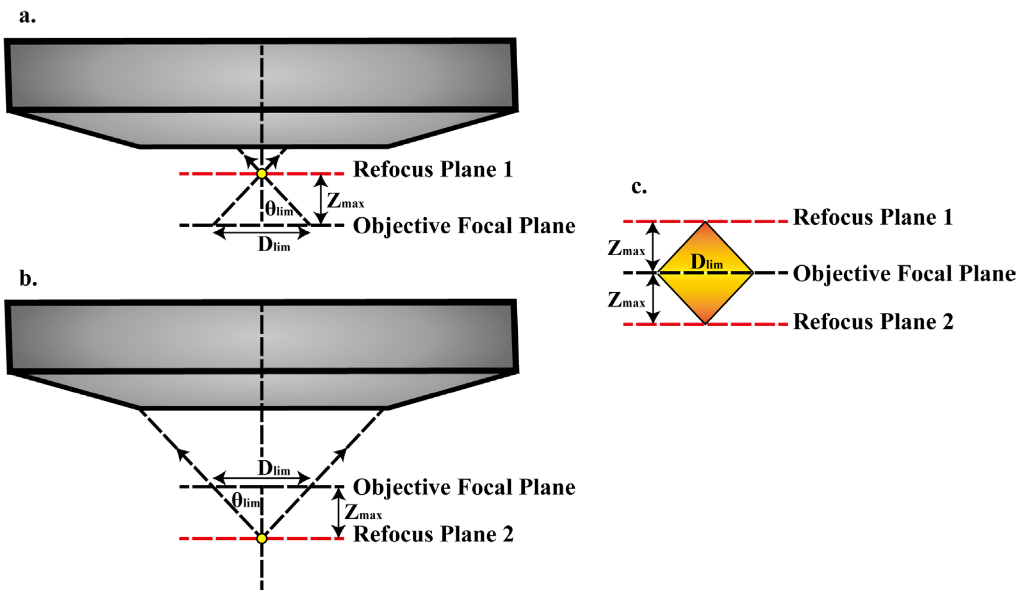

Fig. S7. Schematic showing the geometric maximum refocusing distance from the objective focal plane Z_{max}. (a) shows the closest refocus plane. (b) shows the furthest refocus plane. D_{lim}, limiting FOV of refocus system; θ_{lim}, limiting angular acceptance of the system; Z_{max}, distance at which geometric FOV shrinks to zero. (c) shows the side projection of the geometric maximum refocusing volume.

4. Supplemental Tables

Table S1. Lateral and axial magnifications in all tested cases.

Case	Lateral Magnification (FOV centre)	Axial Magnification	Abs (Lateral Magnification - Axial Magnification) *
Estimated random error	±0.0005	±0.0050	
Maximum estimated systematic error	±0.0005		
Initial	1.3324	1.334	0.0016
O1 (R)	1.3332	1.329	0.0039
O1 (L)	1.3334	1.325	0.0087
O2 (L)	1.3360	1.329	0.0067
O2 (R)	1.3308	1.324	0.0068
TL2 (L)	1.3330	1.325	0.0083
TL2 (R)	1.3342	1.325	0.0095
TL2 (H)	1.3320	1.327	0.0053
TL2 (V)	1.3307	1.315	0.0160
TL2 (I)	1.3323	1.315	0.0176
TL2 (D)	1.3377	1.343	0.0056

*: Absolute value.

Table S2. Gradients of measured lateral magnification lines in all tested cases and gradients of simulated lateral magnification lines in Fig. S6.

Case	Gradient of Lateral Magnification Line (μm^{-1}) $\pm 8\times10^{-6}$	Gradient of Simulated Lateral Magnification Line (μm^{-1})
Initial	2×10^{-5}	-1×10^{-8}
O1 (R)	6×10^{-5}	9×10^{-5}
O1 (L)	-1×10^{-4}	-9×10^{-5}
O2 (L)	4×10^{-4}	3×10^{-4}
O2 (R)	-3×10^{-4}	-3×10^{-4}
TL2 (L)	-2×10^{-5}	-1×10^{-8}
TL2 (R)	1×10^{-6}	-1×10^{-8}
TL2 (H)	2×10^{-5}	
TL2 (V)	3×10^{-5}	
TL2 (I)	4×10^{-5}	
TL2 (D)	-2×10^{-6}	

Table S3. Mean absolute lateral and axial distortions and diffraction-limited volume in all tested cases.

Case	Mean absolute lateral distortion* (μm)	Mean absolute axial distortion** (μm)	Diffraction-limited volume ($\times 10^6$ μm^3)	Percentage difference in diffraction-limited volume from Initial*** (%)
Initial	0.31	0.80	9.6	0
O1 (R)	0.28	0.24	3.4	-65
O1 (L)	0.38	1.21	3.1	-68
O2 (L)	1.04	0.77	2.7	-73
O2 (R)	1.01	1.49	2.7	-72
TL2 (L)	0.17	0.54	2.4	-75
TL2 (R)	0.13	0.85	7.8	-19
TL2 (H)	0.30	0.68	1.3	-87
TL2 (V)	0.49	1.09	0.6	-93
TL2 (I)	0.13	0.47	8.4	-12
TL2 (D)	0.17	0.74	2.7	-72

*: Average over 2D histograms shown in panels c, g, k of figures 2, 3, 4, 5 and S4.
**: Average over 2D histograms shown in panels d, h, l of figures 2, 3, 4, 5 and S4.
***: Percentage Difference from Initial = (Diffraction-limited volume - Diffraction-limited volume of Initial)/(Diffraction-limited volume of Initial)×100.

Table S4. Volume where normalised bead integrated energy is ≥ 0.8 in all tested cases.

Case	Volume where normalised bead integrated energy is ≥ 0.8 ($\times 10^6$ μm^3)	Percentage difference in volume from Initial* (%)
Initial	7.1	0
O1 (R)	9.3	31
O1 (L)	5.7	-20
O2 (L)	6.8	-4
O2 (R)	4.5	-36
TL2 (L)	9.4	32
TL2 (R)	7.3	3
TL2 (H)	1.8	-75
TL2 (V)	2.1	-70
TL2 (I)	9.4	32
TL2 (D)	7.0	-1

*: Percentage Difference from Initial = (Volume without energy loss - Initial volume without energy loss)/(Initial volume without energy loss)×100.

References

1. J. Braat, P. Török, *Imaging optics* (Cambridge University Press, 2019), pp. 582 - 656.
